# The Crosstalk Between Liver Sinusoidal Endothelial Cells and Hepatic Microenvironment in NASH Related Liver Fibrosis

**DOI:** 10.3389/fimmu.2022.936196

**Published:** 2022-06-28

**Authors:** Wei Du, Lin Wang

**Affiliations:** Department of Hepatobiliary Surgery, Xi-Jing Hospital, The Fourth Military Medical University, Xi’an, China

**Keywords:** liver sinusoidal endothelial cells, nonalcoholic steatohepatitis - NASH, liver fibrosis, cross talk, targeted therapy

## Abstract

Chronic liver injury can be caused by many factors, including virus infection, alcohol intake, cholestasis and abnormal fat accumulation. Nonalcoholic steatohepatitis (NASH) has become the main cause of liver fibrosis worldwide. Recently, more and more evidences show that hepatic microenvironment is involved in the pathophysiological process of liver fibrosis induced by NASH. Hepatic microenvironment consists of various types of cells and intercellular crosstalk among different cells in the liver sinusoids. Liver sinusoidal endothelial cells (LSECs), as the gatekeeper of liver microenvironment, play an irreplaceable role in the homeostasis and alterations of liver microenvironment. Many recent studies have reported that during the progression of NASH to liver fibrosis, LSECs are involved in various stages mediated by a series of mechanisms. Therefore, here we review the key role of crosstalk between LSECs and hepatic microenvironment in the progression of NASH to liver fibrosis (steatosis, inflammation, and fibrosis), as well as promising therapeutic strategies targeting LSECs.

## Introduction

Nonalcoholic fatty liver disease (NAFLD) is a spectrum of liver disorder closely related to insulin resistance, type 2 diabetes and genetic susceptibility, including simple fatty liver (SFL), nonalcoholic steatohepatitis (NASH) and related fibrosis/cirrhosis. Histologically, NAFLD is defined as the presence of more than 5% hepatocytes steatosis without evidence of hepatocellular injury (1), while NASH is defined as the presence of more than 5% hepatocytes steatosis and inflammation with hepatocytes injury, with or without fibrosis (2). It is estimated that nearly a quarter of the world’s population suffering from NAFLD, including nearly 100 million in the United States (3). With the global trend of obesity and related metabolic syndrome, NAFLD has become an important cause of chronic liver disease in developed countries such as Europe and the United States. At least 20%-30% of patients with NAFLD develop NASH, the advanced stage of NAFLD, which is emerging as a leading cause of progressive liver fibrosis and end-stage liver disease. Over time, NAFLD and NASH may progress to cirrhosis, with a greater proportion of patients with NASH (20%) developing cirrhosis in their lifetime. In Europe and the United States, NASH is currently the main cause of liver disease in adults waiting for liver transplantation, and it will become the most common indication for liver transplantation in the next decade. Patients with NASH develop hepatocellular carcinoma at significantly higher rates than the general population and have an annual rate that is 12 times higher patients with NAFLD (5.77 vs 0.44 events per 1000 person-years). NASH is a heterogeneous condition with varying rates of disease progression and clinical outcomes, which might be driven by the varying predominant mechanisms for the development of the disease (4). Patients with noncirrhotic NASH are at increased risk even though hepatocellular carcinoma usually occurs in the context of cirrhosis (3). Although the incidence rate and severity of NASH are very high, there is no approved treatment at present. The existing treatment methods are only aimed at controlling related diseases. Therefore, it is very urgent to understand the mechanism of NASH, especially how simple steatosis develops into NASH and then progresses to liver cirrhosis and/or liver cancer (5).

Recently, emerging evidence suggests that intercellular crosstalk rather than a single cell type regulate NASH progression. As the gatekeeper of hepatic microenvironment, LSECs can trigger steatosis, inflammatory response, fibrogenesis *via* communicating with surrounding sinusoidal cells. In the progression of NASH to liver fibrosis, the crosstalk between LSECs and hepatic microenvironment is very complex but important, understanding of which is critical for developing novel therapeutic strategies based on LSECs. In this review, we summarize the intercellular crosstalk between LSECs and surrounding cells in NASH to liver fibrosis, and some potential LSECs targeted therapeutic strategies will be discussed.

## The Intercellular Crosstalk of LSECs in Liver Physiological Microenvironment

**Figure 1:**
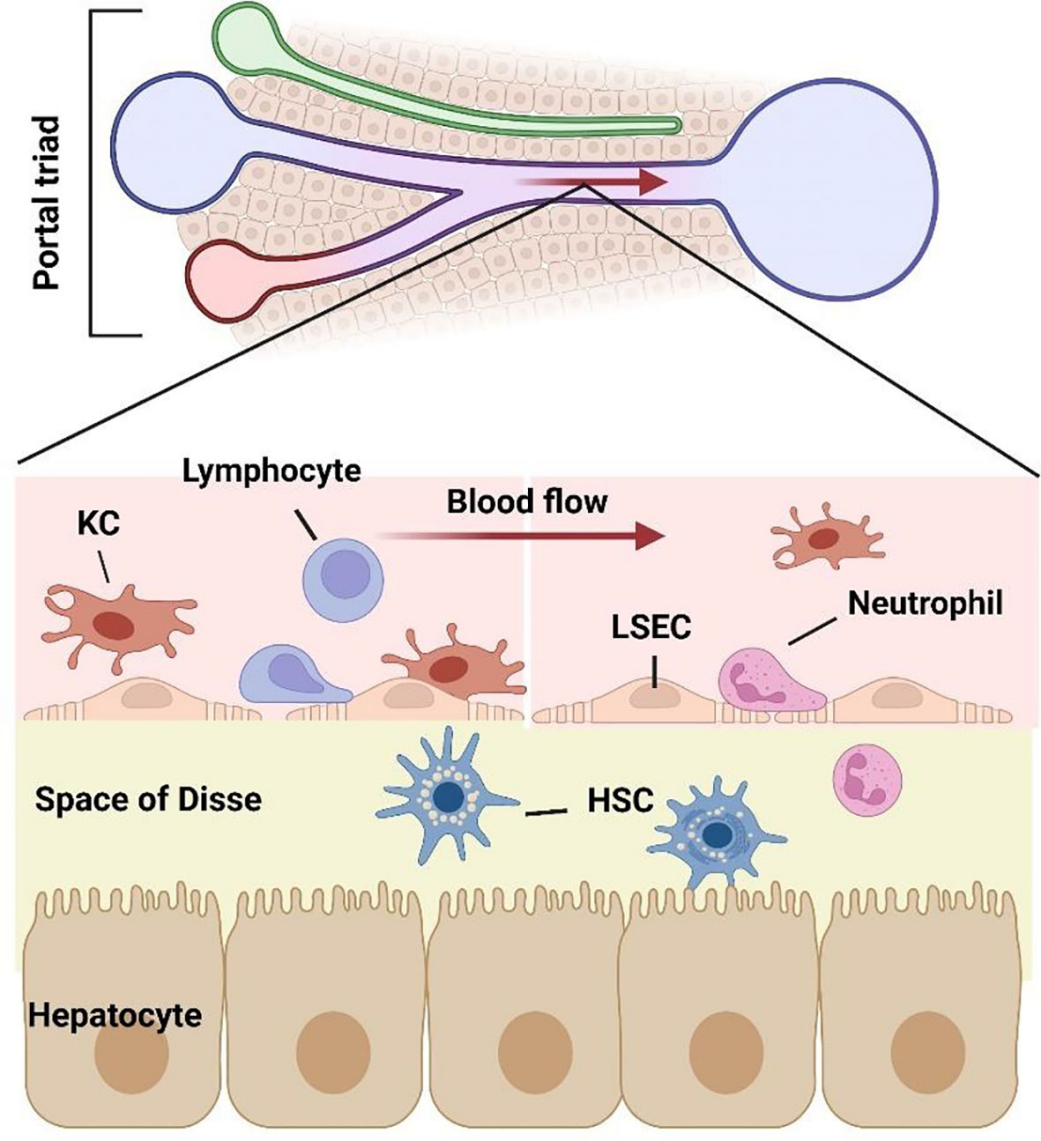
Structure of liver sinusoidal microenvironment. As the gatekeeper of hepatic sinusoidal microenvironment, LSECs constitute the interface between the sinusoid and blood flow. The intercellular crosstalk between LSECs and various cells including hepatocytes, lymphocytes, neutrophils, macrophages, and hepatic stellate cells, which together consist of the hepatic microenvironment.

Liver lobules, as the basic structural and functional unit, are composed of parenchymal cells and non-parenchymal cells. Hepatocytes (HCs) are the primary component of hepatic lobules. HCs constitute 60% of the number and 80% of the volume of hepatic lobular cells, they are the main executors of the liver participating in various physiological functions (6). HCs are distributed radially and form a structure named “liver plate”. HCs have a large number of Golgi bodies, mitochondria and rough endoplasmic reticulum, which play a key role in the process of energy metabolism, material conversion and protein synthesis (7). HCs have strong regeneration capacity and play an important role in liver regeneration after injury (8). Many kinds of nonparenchymal cells (NPCs) are distributed in hepatic sinusoids, constitute 35% of the number and 17% of the volume of liver cells, consist of liver sinusoidal endothelial cells (LSECs) (50%), Kupffer cells (KCs) (20%) and stellate cells (HSCs) (<1%) (9). The remaining NPCs are composed of lymphocytes (25%) and biliary cells (5%) (6). Although NPCs have no advantage in quantity, there is no doubt about their importance to the liver microenvironment (10) (**Figure 1**).

Among the liver NPCs, the most abundant cell type is LSECs. Second only to hepatic parenchymal cells, LSECs constitute 15%-20% of the number of hepatic cells and 3% of the volume of the liver, while account for 50% of NPCs. LSECs are highly specialized endothelial cells with fenestrae, which traverse through the cytoplasm without basement membrane. The fenestrae are 100-150 nm in size and are clustered in groups that have been termed “sieve plate” (11). Fenestration is not a unique structure of LSECs, but also exists in other organs. In mammals, only glomerular endothelial cells and LSECs have open fenestrae, but the glomerular endothelial cells differ from the LSECs in that it locates on organized basement membrane, so LSECs have a unique phenotype in mammals. The fenestration pattern of LSECs in liver lobules vary with zonation, with larger but fewer fenestrae per sieve plate in the periportal region and smaller but more fenestrae per sieve plate in the pericentral region (12). Aging and hypoxia could regulate the capillarization pattern (13). The unique structure of LSECs makes it the most permeable endothelial cell in mammalian vascular system (14). Under different stimuli, LSEC regulates the bidirectional transport of substances between hepatocytes and perisinusoidal space by adjusting the size and number of fenestrae (15, 16). LSECs clear antigens, cell fragments and immune complexes through endocytic vesicles and receptor-mediated endocytosis (17, 18).

Another unique characteristic of LSECs is their expression of high levels of several scavenger receptors compared with conventional endothelium. These receptors on LSECs membrane endow LSECs with high endocytosis capacity, which include scavenger receptor (SR-A, SR-B and SR-H), mannose receptor and Fc gamma-receptor IIb2 (12, 19). The main SRs of LSECs refer to SR-H/stabillin-1 and SR-H/stabillin-2. The SRs is the primary scavenger receptor on the LSECs, mediate endocytosis of polyanionic molecules, including oxidized low-density lipoproteins, hyaluronan, chondroitin sulfate, formaldehyde treated serum albumin, procollagen type I and III N-terminal peptides and advanced glycation end products (20). The mannose receptors are not unique to LSECs and binds to a variety of glycoproteins and microbial glycans, mainly clears circulating collagen alpha chains (I, II, III, IV, V, XI), recruited tissue plasminogen activator regulating fibrinolytic activity and lysosomal enzymes for further use by LSECs. While Fc gamma-receptor IIb2 expressed by LSECs mainly cleans circulating immune complexes formed with IgG, mediating vascular immunity of LSECs (19).

What’s more, LSECs is of great significance for the maintenance of system immune homeostasis (21). LSECs reside along liver sinusoids and separate passenger leukocytes from hepatocytes within sinusoids, further act as a platform for various immune cell populations to lodge in the sinusoidal microenvironment, such as leukocytes, macrophages and lymphoid cells (22). LSECs have vital physiological and immunological functions more than a physical barrier, including filtration, endocytosis, antigen presentation and leukocyte recruitment (23).As the first site of constant exposure to microbial and food antigens derived from the gastrointestinal tract *via* the portal vein, LSECs and KCs play a key role in taking up and cleaning soluble antigens within the hepatic sinusoids (23). It’s necessary to ensure that damaging immune responses are not precipitated against harmless antigens while eliminating invading pathogens simultaneously (23). The initial key step in immune response is the innate pathway of antigen uptake by pattern recognition receptors (24). Pattern recognition receptors mainly expressed on LSECs, are highly evolutionarily conserved and include the Toll-like receptor (TLR) family and scavenger receptors (24). *In vitro*, a variety of TLRs expressed in LSECs also mediated strong inflammatory responses upon ligand stimulation (25, 26). Both of KCs and LSECs constant exposure to lipopolysaccharide (LPS) leads to an LPS-refractory state in LSECs specifically, LPS exposure is associated with reduced nuclear translocation of nuclear factor-κB (NF-κB) and subsequent reduced leukocyte adhesion, which prevents the liver from being a constant exposure to bacterial products from the gut (27, 28). A recent study demonstrated that a high-cholesterol diet exacerbates acetaminophen-induced and liver injury *via* a TLR9/inflammasome-depend manner (28). LSECs not only regulate innate immune responses but also directly regulate adaptive immune responses through antigen presentation to T cells. LSECs can directly contribute to inhibition of effector function of activated T cells. LSECs also express C-type lectin receptors such as L-SIGN and LSECtin not only scavenger receptors, which may contribute to the clearance of pathogens from circulation (29). As an endogenous ligand for LSECtin, CD44 is expressed on activated T cells. LSECtin binding to CD44 leads to inhibition of T-cell activation, proliferation and effector function, this interaction controls local T-cell activation and effector function (30). LSECs express major histocompatibility complex (MHC) class I and II molecules (23). LSECs cross-present soluble antigen to CD8+ T cells on MHC I by using scavenger receptors (31). While they present antigens to CD4+ T cells *via* MHC II-restricted antigen presentation and promote the development of regulatory T cells (32), these tolerogenic properties of LSECs may control autoimmunity in many *in vivo* studies (33, 34).

HSCs are pericytes, located in the space of Disse and surrounded by HCs and LSECs, are the main source of extracellular matrix (ECM) in the liver (35). A single stellate cell can wrap up to four blood sinusoids and alter its structure and function through interactions with surrounding cells (12). HSCs are the predominant cell type leading to liver fibrosis, the injury of LSECs can transform quiescence HSCs into myofibroblast like cells (activated HSCs) (36). The activities of HSCs mainly depend on the interactions with surrounding cells in liver sinusoids (37–39). LSECs is the main source of endothelial nitric oxide (NO), an important substance regulating vascular tension, produced by endothelial nitric oxide synthase (eNOS) (12). At least in part, HCs and HSCs regulate LSEC phenotype *via* paracrine secretion of vascular endothelial growth factor (VEGF) (9). Hepatic macrophages mainly include Kupffer cells resident in the liver and macrophages derived from circulating monocytes (38). Hepatic macrophages, together with surrounding cells, participate in inflammatory response, fibrogenesis and vascular remodeling, are very important to hepatic and systematic response to pathogens (40, 41). Moreover, LSECs also express a variety of adhesion molecules, influence the interaction among sinusoidal cells, are regulated by inflammatory cytokines, including ICAM-1, VCAM-1 and selectin (42). In liver, the space of Disse is filled with ECM (43), which is considered to be the storage place of growth factors, cytokines and some proteins that can be released when needed, promoting the intercellular crosstalk among different types of sinusoidal cells (44). As the gatekeeper of liver sinusoidal microenvironment, LSECs play a central role in liver sinusoidal crosstalk network due to their unique structure and function.

The intercellular crosstalk within the sinusoids is critical to hepatic cell growth, proliferation, migration, differentiation and the maintenance of cell phenotype. In NASH, lipotoxicity induced by excessive accumulation of lipids in HCs upon metabolic imbalance, which promotes the occurrence of oxidative stress and ER stress, metabolic inflammation, hepatocyte ballooning and cell death, and leads to the initiation and progress of fibrosis through the complex crosstalk of sinusoidal cells (45). Understanding the intercellular crosstalk in sinusoids is crucial to better understand the progress of NASH to liver fibrosis, regulating of which may lead to the improvement of the diseases.

## Sinusoidal Crosstalk in NASH Related Fibrosis

The underlying mechanisms of NASH to liver fibrosis are still not clear, multiple pathways involve in lipid accumulation, cellular infiltration and fibrosis. There is a series of key events in the progression from NASH to liver fibrosis, which can be summarized by some hypotheses. Initially, “two hit” hypothesis was established to described the progression of NAFLD (46). In this theory, “first hit” usually refers to the accumulation of lipids, including triglycerides, free fatty acids (FFAs) and cholesterol accumulated in hepatocytes, which leads to NAFLD. In NAFLD, a series of injuries such as lipotoxicity, mitochondrial injury, redox imbalance and inflammation in the liver constitute the “second hit” for NAFLD to develop into NASH (47, 48). The currently accepted theory, “multiple-hit hypothesis”, suggests that there are multiple synergistic events leading to liver inflammation, which may act parallel (49). In this theory, inflammation is not necessarily accompanied by lipids accumulation. On the contrary, it is also possible that inflammation caused by different injuries may exist before steatosis and may promote its progression in NASH. Insulin resistance, oxidative stress, endoplasmic reticulum stress, inflammatory mediators from adipose tissue, dietary factors, gut-liver axis and some epigenetic factors are considered to be the multiple hits for the progression of NAFLD to liver fibrosis (5, 50). Moreover, Type 2 diabetes (T2D) is an important risk factor for the development of NAFLD, then promotes the development of liver injury from simple steatosis to NASH and then leads to liver fibrosis (4, 51).

**Figure 2:**
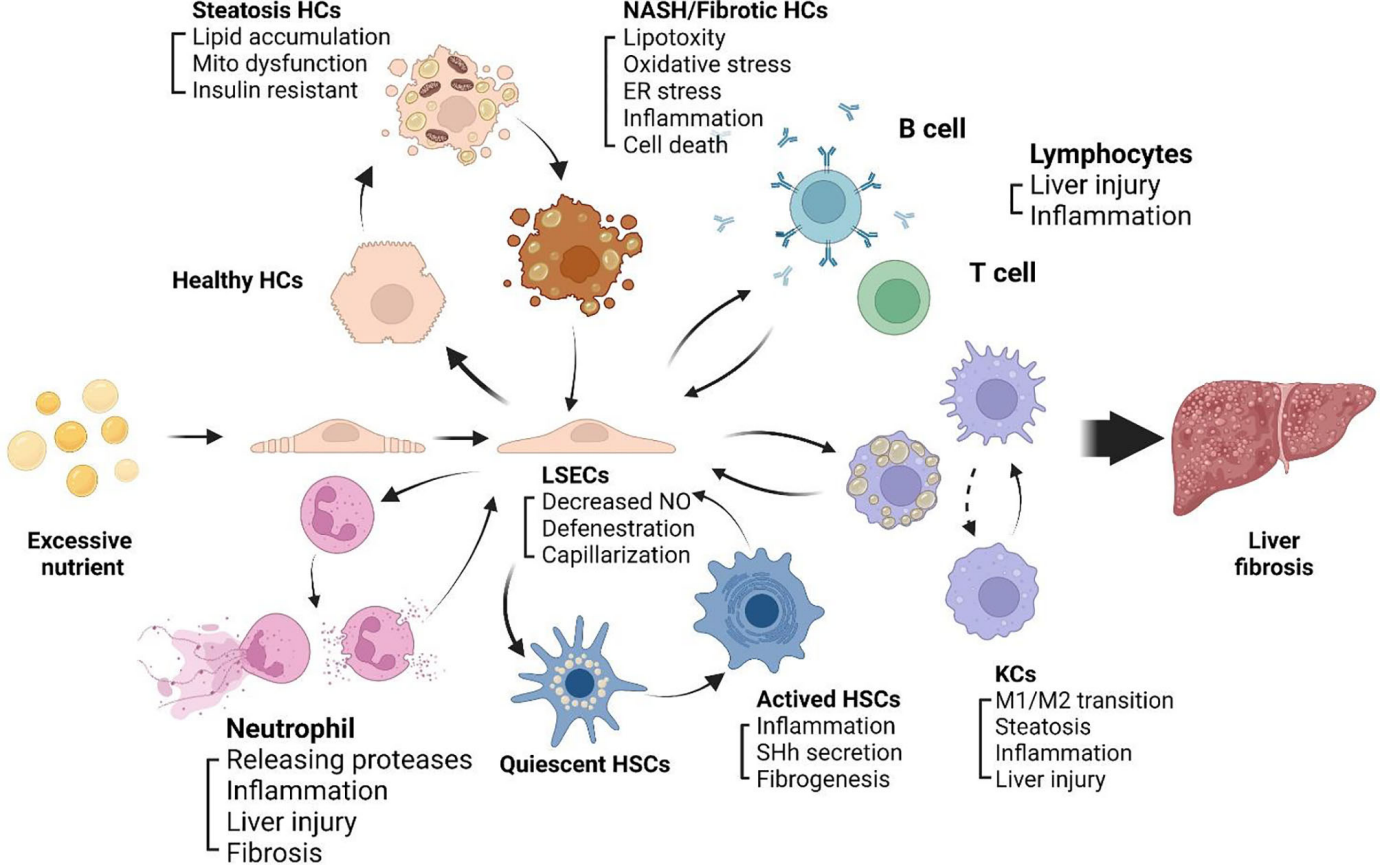
Sinusoidal crosstalk mediated by LSECs play a key role in progression of NASH to liver fibrosis. A series of pathophysiological processes from NASH to liver fibrosis are mediated by LSECs. Capillarization and dysfunction of LSECs appear in early stage of NASH. Capillarized LSECs acquire a pro-inflammatory phenotype, recruiting immune cells including neutrophils, monocytes and lymphocytes to the hepatic microenvironment, promoting HCs steatosis and cell death, activating HSCs and KCs, and promoting liver fibrosis.

The progression of NASH to fibrosis is always accompanied by chronic inflammation, LSECs play a key role in inflammatory response (52). LSECs plays an anti-inflammatory role in the early development of NAFLD by reducing the secretion of proinflammatory chemokines. In NASH, impaired autophagy of LSECs enhance the expression of chemokines, cytokines and adhesion molecules, promote the development of liver inflammation, endothelial-to-mesenchymal transition and liver fibrosis (53). After liver injury, LSECs rapidly lose their highly specialized phenotype and become capillarization, which impairs filtration and endocytosis of LSECs (54). Capillarization refers to the disappearance of the fenestrae and the formation of continuous basement membranes, which transforms LSECs into nonspecific endothelial cells. The accumulation of extracellular matrix (ECM) in liver, which leads to progressive fibrosis. The main mechanism leading to liver fibrosis is a long-standing wound healing process (55), fibrogenesis is driven by dysfunctions of different kinds of sinusoidal cells, including stressed or injured hepatocytes, activated macrophages and HSCs (56). Due to the special position and role of LSECs in the hepatic sinusoids, LSECs can be regarded as the gatekeeper of the hepatic sinusoidal microenvironment, which may mediate the alterations of the hepatic sinusoid microenvironment. Capillarization of LSECs is a key step in the development of chronic liver disease, maintaining normal LSECs phenotype and function can inhibit the development of NASH to liver fibrosis. The intercellular communications among sinusoidal cells involves a series of complex mechanisms, here we review the crosstalk between LSECs and neutrophils, lymphocytes, HCs, KCs, and HSCs within hepatic sinusoids (**Figure 2**).

### Intercellular Crosstalk Between LSECs and Neutrophils

Liver-infiltrating immune cells, with neutrophil infiltration as a hallmark of NASH, play a critical role in the progression of NASH to liver fibrosis (57). Infiltration of neutrophils is commonly observed in patients with NAFLD, and severity of infiltration is associated with disease progression (42). Neutrophils are the first type of immune cells that respond to inflammatory changes in various tissues, including liver, establishing the first line of defense through multiple mechanisms such as phagocytosis, cytokine secretion, reactive oxygen production and neutrophil extracellular trap formation (57, 58). Many studies have revealed that excessive activation of neutrophils induces liver damage within sinusoids, mainly through release of proteases, including myeloperoxidase (MPO), neutrophil elastase (NE), proteinase 3, cathepsins, and matrix metalloprotease (MMP)-9 (42). Elimination of MPO, NE, or proteinase 3 expression or activity *via* genetic or pharmacological approaches may improve pathological changes in NASH (58–60). In inflammatory liver diseases, LSECs influence the composition of hepatic immune populations by mediating diapedesis of leukocyte subsets *via* distinct combinations of adhesion molecules and chemokines. During NAFLD progression, LSECs acquire a pro-inflammatory phenotype and functions, capillarization and dysfunctions of LSECs deteriorate liver inflammation (61). In NASH, LSECs overexpress progressively adhesion molecules including intercellular adhesion molecule-1 (ICAM-1), vascular cellular adhesion molecule-1 (VCAM-1) and vascular adhesion protein-1 (VAP-1) (62), and also produce a number of pro-inflammatory mediators including tumor necrosis factor alpha (TNF alpha), interleukin 6 (IL-6), IL-1 and chemokine ligand 2 (CCL2). *In vivo* and *in vitro* studies showed reduced leukocyte adhesion to hepatic sinusoids when these adhesion molecules are blocked (62, 63). The role of LSECs in initiating immune responses and contributing to progressive liver diseases makes them a potential therapeutic target for treating inflammatory liver diseases. There is an emerging concept that neutrophils can be functionally divided as either N1 or N2, mirroring the M1/M2 and Th1/Th2 classifications. But the precise mechanism of how LSECs induce N1/N2, and their role in NASH and liver fibrosis, are still unknown.

### Intercellular Crosstalk Between LSECs and Lymphocytes

In both humans and rodents, NASH is characterized by B cell and T cell infiltration of the liver as well as by the presence of circulating antigens targeting originating from oxidative stress (64). LSECs regulate the behavior of lymphocytes under both physiological and pathological conditions. The balance of immune subsets determines the progression and outcome of immune responses within the liver, there is now evidence that T cell subsets utilize distinct combinations the mediators to migrate through the hepatic sinusoids under specific microenvironment (23), including α4β1 (65), stabilin1, ICAM1, VAP1 and so on (66). The normal liver is characterized by immunologic tolerance. LSECs block adaptive immunogenic responses to antigens and induce the development of regulatory T cells (67). The progression of NASH to liver fibrosis is associated with intense intrahepatic inflammation and disordered hepatic immunity (68, 69). While under inflammatory conditions, LSECs express high levels of Delta-like and Jagged family of Notch ligands and induce the expression of Notch target genes in Th1 cells, thereby increasing the expression of IL-10 in Th1 cells to exert anti-inflammatory effect (11). However, more studies found that LSEC acquire enhanced immunogenicity in fibrosis, leading to intensified inflammatory microenvironment and altered intrahepatic immunity. For instance, after fibrotic liver injury from hepatotoxins, LSECs become highly proinflammatory and secrete a series of cytokines and chemokines. LSECs gain enhanced capacity to capture antigens, induce the immunogenic T cell to enhance endogenous CTLs and drive potent *de novo* CTL responses (17). Although limited, emerging evidences suggest that B cells participate in the progression of NASH to liver fibrosis (70). Consistently, B cells have been shown to directly contribute to the progression of inflammation and fibrosis in mouse models of NASH and hepatotoxicity (64). However, the specific mechanisms of crosstalk between LSECs and B cells remain unclear (71). In addition, LSECs also express CXCL16, which is a cell membrane-bound ligand for CXCR6, to regulate the number of NKT cells that patrol as part of intravascular immune surveillance in hepatic sinusoids (72).

### Intercellular Crosstalk Between LSECs and HCs

Lots of *in vivo* and *in vitro* studies suggest that, high levels of lipids (73), carbohydrates and gut microbiota products in diet (74, 75), can promote the capillarization and fenestrae loss of LSECs in the early stage of NAFLD (55, 61), while the capillarization of LSECs will aggravate hepatocyte steatosis (76). The capillarization of LSECs reduces the substances exchange between sinusoids and blood, hinders the outward flow of hepatocyte derived very low-density lipoprotein (VLDL), and leads to the retention of cholesterol and triglycerides in the liver (77). Meanwhile, it can also reduce the transfer of chylomicrons to hepatocytes, enhance *de novo* lipogenesis and compensatively increase the production of cholesterol and triglyceride synthesis in hepatocytes (78).

Healthy LSECs secrete a constant compositional level of NO in response to normal stimuli, such as shear stress and VEGF. NO produced by eNOS maintains liver homeostasis and the quiescence of HSCs and KCs (79). LSEC vascular dysfunction can occur in the early stage of NAFLD, before liver inflammation and fibrosis (80). Its dysfunction is mainly manifested in the obstruction of eNOS activation and the decrease of the synthesis level of hepatic vasodilator NO. The main mechanisms leading to this result include high lipid exposure, insulin resistance and intestinal flora imbalance (81). LSECs vascular dysfunction will also reversely promote the development of liver steatosis: first, in the eNOS-/- mouse model, the synthesis of NO is blocked, and the animal liver shows a significant tendency of steatosis (82). Secondly, NO plays a role in regulating hepatic fatty acid synthesis, which has been shown to directly regulate the tricarboxylic acid cycle by limiting citric acid synthesis in mitochondria, inhibiting acetyl CoA and activating AMP-activated protein kinase (AMPK) and other pathways (83, 84). Capillarization reduces LSECs permeability, thus affecting the lipoprotein secretion of HCs and the de nova lipogenesis in HCs. Accumulation of lipid or decreased lipid clearance in liver lead to hepatic steatosis, the abnormal accumulation of lipids in HCs will also lead to reactive oxygen species (ROS) overproduction, mitochondrial respiration injury and endoplasmic reticulum stress, which aggravate the damage of HCs (85).

After sustained liver injury, liver fibrosis develops gradually. More and more evidences show that the interaction between LSECs and HCs plays an important role in the initiation and development of liver fibrosis (86). LSECs and HCs also communicate with each other through the VEGF-A/VEGFR-2 (VEGF receptor 2) signaling in fibrotic liver (87). CD147 is a transmembrane glycoprotein and widely expressed on the surface of various cells including HCs and LSECs, has been proven to be involved in multiple biological process, such as immune response, tumor progression and tissue repair (88). It has been demonstrated that CD147 also play a pivotal role in the angiogenesis of LSECs and simultaneously expressed in HCs and LSECs in fibrotic liver. Interestingly, anti-CD147 antibody inhibits angiogenesis *via* VEGF-A/VEGFR2 axis, thereby improving the process of liver fibrosis (89). In addition, the combination of leukocyte cell-derived chemokine 2 (LECT2) produced by HCs and Tie1 expressed by LSECs also participates in the progress of liver fibrosis. LECT2 is a 16-kDa secreted protein (90), which is a functional ligand of Tie1, a poorly characterized endothelial cell specific orphan receptor. Recently, emerging evidences indicates that LECT2 is involved in many pathological conditions, including sepsis, diabetes, systemic amyloidosis, hepatocarcinogenesis and NAFLD (91–93). *In vivo* studies showed that overexpression of LECT2 promotes sinusoidal capillarization and worsens fibrosis (94).

The crosstalk between LSECs and HCs can promote the fibrogenesis reaction, so the characterization of intercellular communications between LSEC and HCs is an important goal to develop the treatment of NASH to fibrosis in the future.

### Intercellular Crosstalk Between LSECs and KCs

Liver macrophage populations comprises the largest proportion (80%-90%) of resident macrophages in the human body (95), mainly consist of two different subsets of cells, including liver-resident KCs and circulating monocyte-derived macrophages (MoMFs) (96). MoMFs are derived from bone marrow hematopoietic stem cells and recruited to the liver from blood circulation (97–99). Liver macrophages are important mediators of liver inflammation and fibrogenesis in the development of NASH (100, 101). In healthy people, KCs are the main immune cells in the liver. In the healthy rodent liver, KCs account for 20% ~ 35% of all NPCs in the liver (102). Most of KCs are distributed in hepatic sinusoids and have the ability of self-renewal. KCs and MoMFs can usually be polarized into two subtypes, including “pro-inflammatory” M1 macrophages and M2 macrophages involved in “immune regulation” *in vitro* (103). The M1 macrophages produce proinflammatory cytokines such as TNF-α, IL-1β, CCL2 and CCL5. In contrast, M2 macrophages secreted a distinct set of mediators including IL-13, IL-10, IL-4 and transforming growth factor-β (TGF-β) (104).LSECs also have the unique function of modulating KC phenotype in the liver. In the normal liver, LSEC-derived Delta-like ligand 4, a Notch ligand, and TGF-β contribute to the maintenance of KC identity (105).

As the gatekeeper of hepatic immunity, LSECs interacted directly with the immune cells and antigens in the blood flow (11). Adhesion of monocytes to LSECs is a crucial step for inflammation response in NASH, which verified the central role of macrophages in the progression of NASH to liver fibrosis. There is a dynamic balance between M1/M2 ratio, and the imbalance of M1/M2 ratio may be the key to the progression of NASH to liver fibrosis. In the early stage of NAFLD, mild inflammation is often controllable and contributes to liver repair and regeneration after injury. LSECs play an anti-inflammatory role by inhibiting KCs activation and monocyte migration. LPS or excess FFAs active KCs to release a large number of pro-inflammatory cytokines (TNF-α, TNF-β and NF-κB etc.) and promote lipid accumulation and oxidative stress response in HCs through paracrine, which drives the progression from simple steatosis to NASH and even fibrosis. As NASH progresses, it has been noted that KCs and MoMFs in NASH liver exhibited a notable shift toward a proinflammatory phenotype on the basis of their gene expression signatures at the single-cell level (39). The number of M1 macrophages increased significantly, while the number of M2 macrophages decreased remarkably (106). And capillarization of LSEC occurs, which is required for activation of KCs (61). LSECs convert to a pro-inflammatory phenotype, producing pro-inflammatory mediators that lead to the activation of KCs (23). Activated KCs participate in angiogenesis by secreting ROS and cytokines including TNF-α, PDGF and platelet activating factor (PAF) (107). The pro-inflammatory phenotype of LSECs increases the expression of the chemokine CCL2, recruiting monocytes to the liver (62). In addition, LSECs in NASH mouse models overexpressed adhesion molecules ICAM-1, VCAM-1 and VAP-1, which are critical for monocyte adhesion, transport, and participation in local inflammatory responses (42, 55). While in the fibrosis model, intercellular crosstalk between KCs and LSECs results in fenestration loss and expression of CD31 increased, a surface marker of LSECs dedifferentiation (108, 109). The precise contribution of LSECs-KCs interactions to the pathogenesis of liver fibrosis is still to be elucidated.

### Intercellular Crosstalk Between LSECs and HSCs

HSCs are the main source of ECM synthesis, distributed in space of Disse. Activation of HSCs is now well established as a central driver of fibrosis in experimental and human liver injury (110, 111). In normal liver, differentiated LSECs prevent activation of HSCs and promote reversion of activated HSCs to quiescence *via* VEGF-stimulated nitric oxide production (112). Chronic injury leads to loss of LSECs differentiation and capillarization, diminishes their ability to suppress HSCs activation (111). Uninterrupted inflammation can cause HSCs to active and differentiate into myofibroblasts. The activated myofibroblasts release a large amount of extracellular matrix (ECM) into hepatic sinusoids, which are rich in collagen fibers, eventually promote liver fibrosis or cirrhosis (113). In the early stage of NASH, free cholesterol accumulation in HSCs sensitizes the cells to TGF-β induced activation through enhancement of Toll-like receptor 4 (TLR4) mediated downregulation of TGF-β pseudo receptor BAMBI (bone morphogenetic protein and activin membrane bound inhibitor) (114). LSECs become capillarized and transform into pro-vasoconstriction, pro-inflammation, pro-angiogenesis and pro-fibrosis phenotypes (115, 116). Intercellular crosstalk between LSECs and HSCs cells is an important driver of liver fibrosis (22). Capillarized LSECs no longer keep HSCs quiescence, but secrete fibronectin (FN), platelet-derived growth factor (PDGF), TGF-β and Hedgehog (Hh) ligands, and reduce the transcription factor Kruppel like factor 2 (KLF2), which is a protective molecule of hepatic vascular endothelium (117), to activate HSCs. Meanwhile, capillarization of LSECs may also lead to impaired blood oxygen diffusion, leading to hypoxia environment, which further induces rapid activation of HSCs and expression of HIF-1α (118). Activated HSCs further act on quiescent HSCs and LSECs through autocrine TGF-β1, forming a positive feedback loop on the progression of liver fibrosis (119). HSCs begin to proliferate, contract and deposit a large amounts of collagen fibers and extracellular matrix molecules in the liver parenchyma, leading to organ stiffening and disrupting all cellular functions (36).

Exosomes also play a bidirectional regulatory role in crosstalk between LSECs and HSCs. Dedifferentiated LSECs secrete exosomes rich in sphingospkinase-1 to promote the activation and migration of HSCs. While activated HSCs can also release Hh-rich exosomes and alter the expression of LSECs gene (120, 121). In addition, C-X-C chemokine 12 (CXCL12)/stromal derived factor-1 (SDF-1) produced by LSECs promotes HSCs migration during chronic liver injury (122). Sustained FGF receptor 1 (FGFR1) activation results in higher CXCR4 expression in LSECs than CXCR7, which stimulates HSCs proliferation and causes liver fibrosis (123). DLL4, a ligand of notch signaling pathway, is highly expressed in LSECs of fibrotic human liver tissues, as well as that from CCl_4_-induced mice. Overexpression of DLL4 accelerates defenestration of LSECs, and also increases the coverage of liver sinusoids by HSCs through endothelin-1 (ET-1) synthesis (124, 125). Meanwhile, ET-1 produced by HSCs plays a key role in the regulation of eNOS activation in LSECs and defenestration of LSECs (126). To clarify the crosstalk between LSECs and HSCs in different stages of chronic liver disease and the mechanism of gene expression and secretion profile alterations of LSECs is of great significance for preventing and reversing NASH to liver fibrosis.

## Targeted Therapeutic Strategy of LSECs in NASH to Liver Fibrosis

Currently, no specific drug has been approved for clinical use to treat patients with NASH or liver fibrosis. As LSECs dysfunction drives the progression of NASH to liver fibrosis, restoring LSECs phenotype and regulating the crosstalk between LSECs and other liver cells within sinusoidal microenvironment are identified as attractive targets for treatment (**Table 1**).

**Table 1:** Therapeutic drugs related to crosstalk between LSECs and other sinusoidal cells involved in this paper.

Target	Drug	Mechanisms	References
adhesion-related molecules	Cenicriviroc TERN-201	ameliorating hepatic inflammation reducing the recruitment of CCR2+ monocyte in the liver inhibiting VAP-1 to control inflammation in NASH	(65, 127)
NO-related signaling	Statins WAY-362450 Praliciguat	ameliorating the LSECs phenotype that improves HSCs status by paracrine manner promoting ADMA degradation and recovery of NO pathway increasing the cGMP level in LSECs	(117, 128–131)
angiogenesis	L1-10 AAV9 with shRNA of LECT2 combined with recombinant VEGF or bevacizumab	preventing sinusoidal capillarization inhibiting the interaction between LECT2 and its receptor Tie1 inhibiting VEGF/VEGFR signaling	(132–134)

### Targeting Adhesion-Related Molecules of LSECs

Adhesion of immune cells to LSECs is an essential step of inflammation in NASH. Adhesion molecules are abnormally expressed on LSECs, which provides multiple potential targets to control inflammation in NASH. Interference with recruiting signals would affect the intercellular communication between the recruited and resident immune cells in the liver. The pro-inflammatory LSECs overexpress adhesion molecules including VAP-1, VCAM-1, CD31, ICAM-1 and E-selectin (16, 55). Blocking these molecules or their ligands may control the development of progression of NASH to liver fibrosis. TERN-201, a kind of potent VAP-1 inhibitor, is still undergoing clinical trials in China for the treatment of NASH (62, 63, 135). Circulating inflammatory monocytes are attracted to the hepatic microenvironment *via* their chemokine receptor C-C motif chemokine receptor 2 (CCR2), while the corresponding CCL2 is strongly expressed by various liver cells such LSECs and KCs (136). Cenicriviroc (CVC), a dual antagonist of CCR2 and CCR5, ameliorates hepatic inflammation in NASH mice models by reducing the recruitment of CCR2+ monocyte in the liver. The drug has been shown to be effective in reducing fibrosis in a Phase III clinical trial in NASH patients (NCT03028740).

### Targeting NO-Related Signaling of LSECs

LSECs are the major producers of NO in the liver (137). The balance of NO is critical in maintaining the morphology and endothelial function of LSECs to keep the quiescence of HSCs and KCs, it also thoroughly participates in the regulation of liver lipid and glucose homeostasis (138). Activation of endothelial Notch in LSECs aggravated the NASH phenotype through eNOS-sGC signaling (139, 140). Thus, targeting NO-related signaling may be an attractive therapeutic strategy. Statins can increase NO bioavailability in the sinusoidal microcirculation through reducing activity of RhoA and enhancing activity of Akt/protein kinase B (PKB) (128, 141). In addition, statins also regulate the LSECs phenotype that paracrinally improves HSCs status (117). Farnesoid X receptor (FXR) is a bile-acid responsive transcription factor that associated inflammation, fibrosis, and vascular homeostasis (129, 142, 143). FXR agonism plays a key role in the recovery of NO pathway and endothelial cells dysfunction, which could effectively increase the expression of eNOS in LSECs by promoting degradation of asymmetric dimethylarginine (AMDA) in bile duct ligation (BDL) rats (144). WAY-362450, an synthetic potent FXR agonist, can protect against NASH and hepatic fibrosis in methionine/choline-deficient (MCD) diet-fed mice model (130). Under oxidative, NO signal and the affinity between NO and sGC are interfered, which leads to the dysfunction of LSECs. Therefore, regulating of sGC activity may be a potential approach to restore the phenotypic changes, prevent sinusoidal capillarization and activation of HSCs. Praliciguat, an oral soluble sGC stimulator with extensive distribution to the liver in clinical development, effectively reduced inflammation, fibrosis, and steatosis by enhancing NO signaling in preclinical NASH models (131).

### Targeting Angiogenesis of LSECs

Capillarization of LSECs occurs in the early stage of NASH, angiogenesis is deeply involved in liver fibrogenesis. Pathological capillarization of LSECs promotes liver steatosis, inflammation, and fibrosis. Though current controversial results suggest it is difficult to treat NASH or liver fibrosis through vascular targeting, there a variety of anti-angiogenic therapies have shown promising results (132). As a functional ligand of endothelial cell-specific receptor Tie1, LECT2 promotes liver fibrosis by inhibiting portal angiogenesis and promoting capillarization of liver sinusoids in various liver fibrosis models. Studies have shown that inhibiting the interaction between LECT2 and its receptor Tie1 effectively improved liver fibrosis by using peptibody L1-10 (133). Liver endothelial cells located in different zonations are heterogeneous, and their changes and roles in the pathological process from NASH to fibrosis are also different. Therapeutics that targeted a single vascular endothelial cell is not enough to treat liver fibrosis effectively. Combined muti-target therapies provide innovative insights for blocking or slowing down liver fibrosis. In a recent study, researchers explored a vascular-targeted therapy for liver fibrosis by using the adeno-associated viral vector serotype 9 (AAV9) with a short hairpin RNA (shRNA) of LECT2 combined with recombinant VEGF (rVEGF, VEGF/VEGFR signaling activator) or bevacizumab (VEGF neutralizing antibody, VEGF/VEGFR signaling inhibitor) to simultaneously regulate hepatic endothelial cells in different zonations (134).

### Nanomaterial-Based Drug Delivery Targeting LSECs

For several decades, researchers have been developing drug carriers that will increase the drug delivery to liver for targeting NASH and liver fibrosis and decrease the side effects of drug metabolism. Nanoparticle such as exosome offer novel insights for NASH and liver fibrosis due to their low immunogenicity, low toxicity and high engineering (145). Exosomes are cell-derived nanovesicles that are involved in the intercellular crosstalk. Therapeutics, such as small molecules or nucleic acid drug, can be incorporated into exosomes and then delivered to specific types of cells or tissues to realize targeted drug delivery (146). Activation of Notch signaling in macrophages mediates the progression of NASH to fibrosis in the liver, study shows that transcription factor decoy oligodeoxynucleotides delivered by exosomes could be taken up by hepatic macrophages and ameliorate hepatic fibrosis by inhibiting Notch signaling in mice with liver fibrosis (41). Engineering exosomes targeting LSECs are is feasible for NASH treatment (41, 146). Liposome, another promising nanomaterial for drug delivery, can encapsulate both hydrophobic and hydrophilic drugs and the hydrophilic membrane shell may be modified by chemical moieties to target specific liver cell type (41, 145). LSECs could be specifically recognized by hyaluronic acid (HA)-based liposomes through the HARE/Stabilin-2 receptor (147, 148). Nanoparticles decorated with a stabillin receptor ligand can target to natural tolerogenic LSECs is able to generate regulatory T cells, which can suppress antigen-specific immune responses. Nanoparticle modified with the peptide of stabillin receptor ligand can target to LSECs, and modified nanoparticles loaded with different drugs have shown therapeutic effects in in a variety of autoimmune disease models in mice (149, 150). What’s more, functional efferocytosis of apoptotic vesicles restore liver macrophage homeostasis and ameliorates lipid metabolism (151). However, there have been no studies of LSECs-targeting modified nanoparticles for NASH or liver fibrosis treatment. The development of nanomedicine offers novel insights for NASH and liver fibrosis therapy.

## Conclusion and Future Perspectives

As the gatekeeper of hepatic microenvironment, LSECs have multiple functions due to their unique structure and anatomical position, including substance exchange and clearance, blood flow regulation, and immune regulation under physiological conditions. In the early stages of NAFLD, lipotoxicity, adipokines, inflammation and gut microbiota derived products trigger LSECs dedifferentiation, driving capillarization and dysfunction of LSECs. In NASH, LSECs can no longer maintain the quiescence of KCs and HSCs, but transform into the phenotype of pro-inflammatory, pro-angiogenic and pro-fibrogenic. The crosstalk among LSECs and other sinusoidal cells plays an important role in the physiological and pathological processes of the liver, thus keeping LSECs healthy has high therapeutic potential for NASH related liver fibrosis.

The pathological process from NASH to fibrosis includes liver steatosis, inflammation, and fibrosis. Here we only review intercellular crosstalk mediated by LSECs within the sinusoidal microenvironment, the detailed mechanisms are involved with multiple alterations of LSECs, including morphology and endothelial function, paracrine and autocrine signals, hepatic cell-derived extracellular vesicles, and autophagy abnormalities. In fact, the crosstalk among various cells in the sinusoid microenvironment is very complex. There are no strongly specific drugs to treat NASH and liver fibrosis, and several candidates are still undergoing preclinical or clinical trials. Currently, our understanding of intercellular crosstalk in the hepatic sinusoidal microenvironment is very limited. In recent years, the rapid development of single-cell technology has provided researchers with powerful tools to gain deep insights into the molecular mechanisms involved in diseases. To explore the intercellular crosstalk between various sinusoidal cells at the single-cell level will help us deeply understand the pathological process from NASH to liver fibrosis, so as to explore better therapeutic strategies.

## Author Contributions

DW designed the outline of the review and drafted the manuscript. WL contributed his scientific advice and revision of the manuscript. All authors read and approved the submitted version.

## Funding

This study was supported by the MOST 2016YFA0102100 (The National Key Research and Development Program of China: Stem Cell and Translational Research), and NSFC 81670863, 81422009, 81401940, 81770560, 81800533.

## Conflict of Interest

The authors declare that the research was conducted in the absence of any commercial or financial relationships that could be construed as a potential conflict of interest.

## Publisher’s Note

All claims expressed in this article are solely those of the authors and do not necessarily represent those of their affiliated organizations, or those of the publisher, the editors and the reviewers. Any product that may be evaluated in this article, or claim that may be made by its manufacturer, is not guaranteed or endorsed by the publisher.
